# A Swollen Inferior Gemellus Muscle with Hematoma Mimics Sciatica—A Case Report

**DOI:** 10.3390/diagnostics12051080

**Published:** 2022-04-26

**Authors:** Co Yih Siow, Kuan-Lin Chen

**Affiliations:** 1 Department of Physical Medicine and Rehabilitation, Changhua Christian Hospital, Changhua 500209, Taiwan; chloescy@gmail.com; 2 Department of Physical Medicine and Rehabilitation, Yuanrung Hospital Yuansheng Branch, Changhua County 510007, Taiwan; 3 Department of Exercise Health Science, National Taiwan University of Sport, Taichung 404401, Taiwan

**Keywords:** inferior gemellus, sciatica, ultrasound, magnetic resonance imaging

## Abstract

Extra-spinal causes of sciatic pain are normally underdiagnosed, as they are extremely uncommon. Although pyriformis syndrome is recognized as one of the main causes of sciatic pain, other pelvic muscles that could cause sciatic pain are often overlooked. The present article describes a swollen inferior gemellus muscle with hematoma initially diagnosed with ultrasonography and later confirmed with magnetic resonance imaging (MRI) scan. Ultrasound revealed a swollen muscle with hematoma between the ischial tuberosity and the medial surface of the greater trochanter of the femur. MRI scan showed edematous change with an increased enhancement of the right inferior gemellus muscle. Ultrasound could be used to diagnose inferior gemellus pathology, but the muscle is easy to miss. Therefore, MRI could be preferred for conditions that impact deep or large areas in confirming inferior gemellus pathology.

## 1. Introduction

Either spinal or extra-spinal causes could cause sciatic pain. Extra-spinal causes of sciatic pain are underdiagnosed due to their rarity. Pyriformis syndrome is probably the most well-known extra-spinal cause of sciatic pain, but due to the lack of literature and general knowledge is often under-recognized. Nevertheless, other pelvic muscles could cause sciatic pain, which is often overlooked [1].

The inferior gemellus muscle is an abductor and external rotator muscle of the hip that stabilizes the head of the femur in the acetabulum. It originated from the upper part of the lateral surface of the ischial tuberosity and the medial surface (intrapelvic origin). The muscle passes through the tendon of the obturator internus and together with the superior gemellus muscle, subsequently inserts into a medial surface of the greater trochanter of the femur [2]. Strain of inferior gemellus muscle has rarely been reported; to our knowledge, there has only been one such case reported in previously published literature [3]. Tear of inferior gemellus muscle is similarly rare [4]. Injury of the inferior gemellus could be caused by movement exceeding the normal range of motion of the hip joint.

Herein, the present article reports a patient suffering from inferior gemellus muscle strain with hematoma after a basketball practice game. Clinical, ultrasonographic, and magnetic resonance imaging (MRI) findings are described to underline the importance of considering the condition in the diagnostic algorithm of hip pain of unknown origin. This case is of considerable clinical value due to its rare clinical, ultrasonographic, and MRI findings.

## 2. Case Presentation

A 57-year-old man with an unremarkable past medical history presented to the outpatient clinic with right gluteal pain. One week before the presentation, the patient had a basketball practice game. During the game, he made sudden lower limb turning movements. A snapping sound originating from his right gluteal area was heard immediately after the movement, and the man reported sudden onset of right-sided gluteal pain radiating towards the right hip area. The pain was only partially relieved by over-the-counter nonsteroidal anti-inflammatory drugs (NSAIDs) to curb inflammation.

On clinical examination, inspection revealed an antalgic gait and inability to hyperextend the right hip. There was local tenderness over the right deep gluteal area, but no swelling or bruising over the right groin, thigh, or buttock was noted. There was no tenderness of the paraspinal muscle, the greater trochanter, the insertion of the hip adductor muscles, or the iliotibial band. The straight leg raise test was positive on the right side. The flexion, adduction, internal rotation tests were positive. Patrick’s test was also positive. However, the springing test of lumbar region was negative.

**Figure 1:**
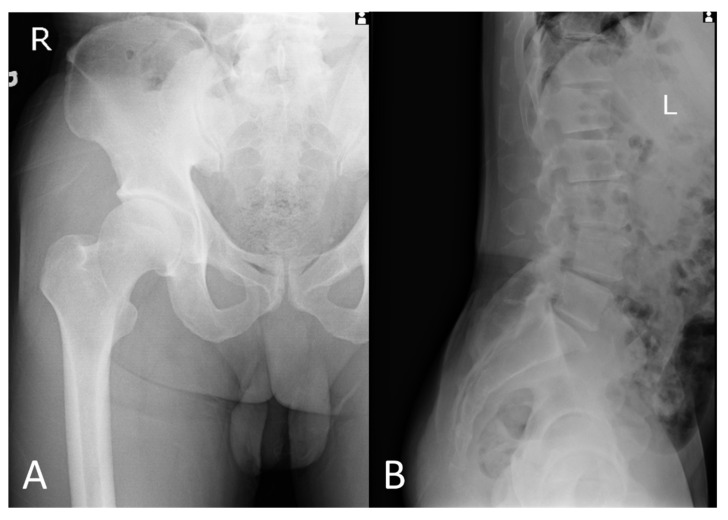
Plain radiographs of hip and lumbar spine. (**A**) Plain radiographs showed a hip with subtle subchondral bone sclerosis, which is compatible with early-stage OA. (**B**) Plain radiographs showed lumbar spines with spondylosis, disc space narrowing, degenerative sclerotic change at the L2/3 level, retrolisthesis at the L2/3, L3/4 level, and grade I spondylolisthesis at the L4/5 level, however not related to this acute pain.

Plain radiographs of the right hip revealed a hip with subtle subchondral bone sclerosis, which is compatible with early-stage OA, as shown in Figure 1A. Plain radiographs of the lumbar spine revealed spondylosis, disc space narrowing, degenerative sclerotic change at the L2/3 level, retrolisthesis at the L2/3, L3/4 level, and grade I spondylolisthesis at the L4/5 level; however, not related to this acute pain, as shown in Figure 1B.

**Figure 2:**
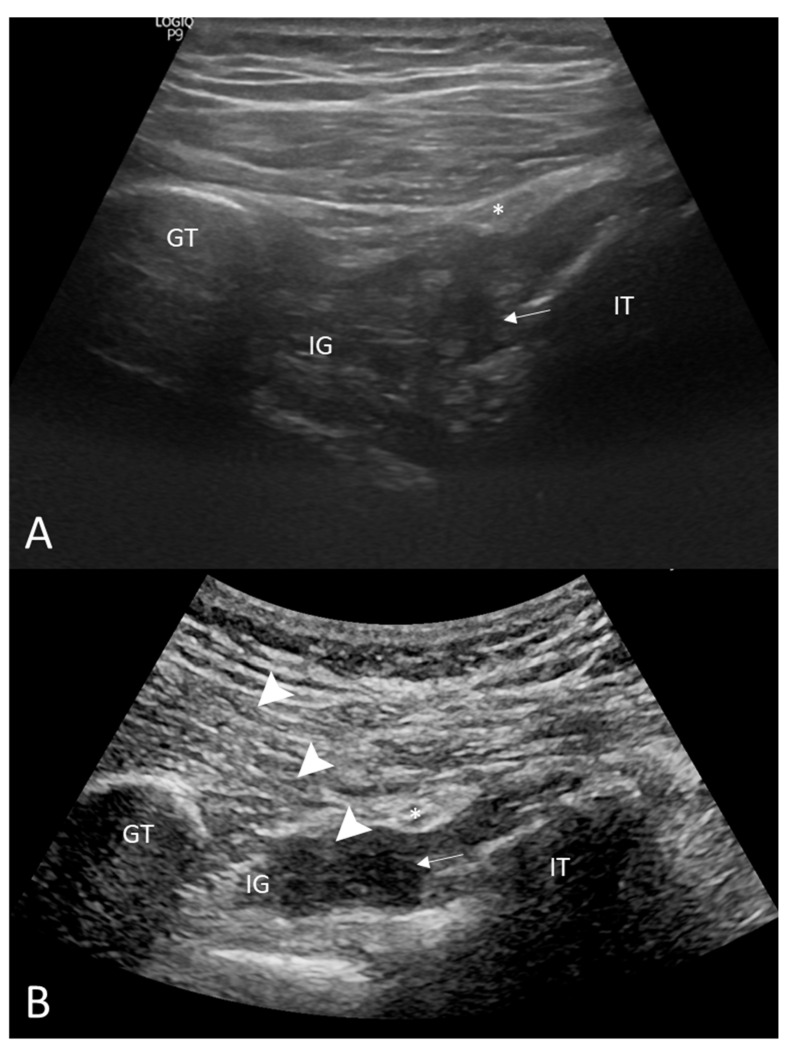
Musculoskeletal sonograph of inferior gemellus. (**A**) Musculoskeletal sonography showed a swollen muscle with hematoma (arrow) between the ischial tuberosity and medial surface of greater trochanter of the femur. Sciatic nerve (asterisk) was superficial to the muscle. The inferior gemellus strain with hematoma was highly suspected before an MRI scan was performed. (**B**) Musculoskeletal sonography showed ultrasound-guided platelet-rich plasma injection (arrowhead) to the inferior gemellus. GT = greater trochanter; IG = inferior gemellus; IT = ischial tuberosity.

**Figure 3:**
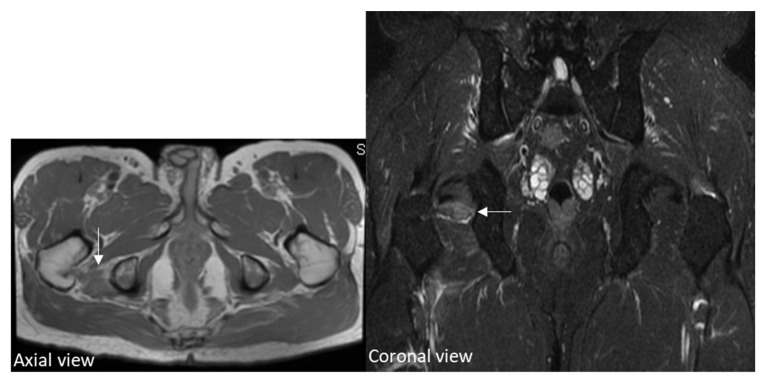
Coronal short tau inversion recovery images and axial proton-density weighted images of hip MRI. MRI scan demonstrated edematous change with an increased enhancement (arrow) of the right inferior gemellus muscle. Therefore, the right inferior gemellus muscle myositis was impressed.

As the severe pain became more frequent, musculoskeletal sonography was performed to identify the cause. The sonography revealed a swollen inferior gemellus with hematoma between the ischial tuberosity and the medial surface of the greater trochanter of the femur, indicating the muscle strain with hematoma as shown in Figure 2A. MRI scan of the right lower limb demonstrated edematous change with an increased enhancement of the right inferior gemellus muscle as shown in Figure 3. Musculoskeletal sonography and MRI indicated the diagnosis of right inferior gemellus muscle myositis.

The patient was treated with an ultrasound-guided steroid injection (Triamcinolone 10 mg mixed with lidocaine 10 mg), also as a diagnostic treatment, followed by oral NSAID (Diclofenac 75 mg divided three times per day for 7 days) and rehabilitation therapies, including hot packing, ultrasound therapy, interferential current, and therapeutic exercises. The pain immediately improved after the steroid injection and local anesthetics. Upon weekly follow-up visits, both the pain and the range of movement of the right hip improved. A half month later after the steroid injection, the patient received ultrasound-guided platelet-rich plasma injection (shown in Figure 2B) per month for 3 times to promote muscle recovery. The patient returned to previous daily activity after 3 times injections and physical therapy.

## 3. Discussion

Inferior gemellus, superior gemellus, and obturator internus muscles are called triceps coxae (i.e., triceps of the posterior hip) and are part of a larger, three-headed muscle complex [5]. The insertion of the inferior muscle is at the medial surface of the great trochanter. Unlike the superior gemellus, which was frequently innervated by the nerve to the obturator internus or the nerve to the quadratus femoris or both, the inferior gemellus was innervated by branches of the quadratus femoris nerve from the anterior or deep surface [2]. Historically, they were regarded as a single muscle with common origin but current evidence suggests them as separate entities with different origins and innervations, therefore surgeon awareness of anatomical variants may help surgeons during surgical intervention.

To perform the sonographic examination of the inferior gemellus muscle, the medial end of the transducer needed to be rotated caudally while maintaining the fixed position at the lateral end. The procedure required the operator to turn the transducer slowly and carefully while making passive rotation of the hip. As the origin of the inferior gemellus muscle was at the ischium (ischial tuberosity), immediately inferior to where the obturator internus passes, misidentification is potential. The heel–toe maneuver could be performed to facilitate the visualization of the ischial attachment of the inferior gemellus muscle [6].

The pathway of the sciatic nerve is deep to the piriformis muscle and superficial to the triceps coxae muscles. If the sciatic nerve is compressed by these muscles, whenever there is inflammation or trauma, it will cause sciatic pain, which needs the differential diagnosis of non-discogenic sciatica [7,8]. The main pitfall associated with the sonographic examination of the inferior gemellus muscle is that the muscle is easy to miss. A previous case report described right sciatica in a 51-year-old female initially diagnosed with piriformis syndrome, but the symptoms persisted despite piriformis injection and piriformis stretching exercises. In that case, MRI showed hyperintensity of the right inferior gemellus and confirmed right inferior gemellus injury [1]. The case demonstrated the value of MRI in identifying other pelvic muscles anatomically near the sciatic nerve as causes of sciatic pain, particularly for cases resistant to treatment. Therefore, MRI is the best choice for evaluating the body for musculoskeletal problems.

No cases of inferior gemellus hematoma had been published previously. Overall, reports of sonographic pathology of inferior gemellus or even superior gemellus were lacking in published literature. However, the patient with gluteal pain from obturator internus tendinitis and bursitis successfully treated with ultrasound-guided injection had been reported [9].

## 4. Conclusions

This article is the first case report describing ultrasound for diagnosing the inferior gemellus problem.

## Data Availability

The datasets during and/or analyzed during the current study are available from the corresponding author on reasonable request.

## Abbreviations

MRI: magnetic resonance imaging; NSAIDs: nonsteroidal anti-inflammatory drugs: femoroacetabular impingement (FAI).
